# Recent Progress of the Vat Photopolymerization Technique in Tissue Engineering: A Brief Review of Mechanisms, Methods, Materials, and Applications

**DOI:** 10.3390/polym15193940

**Published:** 2023-09-29

**Authors:** Ying Li, Xueqin Zhang, Xin Zhang, Yuxuan Zhang, Dan Hou

**Affiliations:** 1 College of Chemistry and Materials Engineering, Beijing Technology and Business University, Beijing 100048, China; 2 FuYang Sineva Materials Technology Co., Ltd., Beijing 100176, China; 3 Chinese Academy of Meteorological Sciences, China National Petroleum Corporation, Beijing 102206, China

**Keywords:** vat photopolymerization, bioprinting, stereolithography, digital light processing, continuous liquid interface production, computed axial lithography, biocompatibility, tissue engineering

## Abstract

Vat photopolymerization (VP), including stereolithography (SLA), digital light processing (DLP), and volumetric printing, employs UV or visible light to solidify cell-laden photoactive bioresin contained within a vat in a point-by-point, layer-by-layer, or volumetric manner. VP-based bioprinting has garnered substantial attention in both academia and industry due to its unprecedented control over printing resolution and accuracy, as well as its rapid printing speed. It holds tremendous potential for the fabrication of tissue- and organ-like structures in the field of regenerative medicine. This review summarizes the recent progress of VP in the fields of tissue engineering and regenerative medicine. First, it introduces the mechanism of photopolymerization, followed by an explanation of the printing technique and commonly used biomaterials. Furthermore, the application of VP-based bioprinting in tissue engineering was discussed. Finally, the challenges facing VP-based bioprinting are discussed, and the future trends in VP-based bioprinting are projected.

## 1. Introduction

3D printing, also known as additive manufacturing, is a revolutionary technology that distinguishes itself from traditional subtractive manufacturing. Under the control of a computer, materials with controlled cross-section geometries can be accumulated layer by layer to form 3D objects with virtually any structure. It is particularly suitable for the fabrication of small-batch production or personalized complex structures [1,2]. Recently, 3D printing has been successfully applied in the fields of regenerative medicine and tissue engineering, which is considered a promising method to address the growing demand for living organ transplantation [3]. 3D bioprinting has been used to fabricate artificial substitutes with biological functionalities to repair, enhance, or maintain tissue functions [4]. Cells, biological factors, and bioactive scaffolds are the fundamental building blocks for tissue engineering [5]. Hydrogels are widely utilized as tissue engineering scaffold materials because of their ECM-like network structures, which can be tailored to possess a range of mechanical, chemical, and biological properties that promote cell adhesion, proliferation, and migration [6].

Compared to traditional fabrication techniques such as freeze-drying, electrospinning, and thermally induced phase separation, 3D bioprinting offers numerous advantages in fabricating artificial tissues: 1. high accuracy in replicating multiscale 3D tissue structures [7]; 2. controlled printing of cell-laden complex 3D structures [5]; 3. capable of constructing multi-network structures to facilitate the transportation of nutrients and oxygen for the regeneration of tissues [8].

According to printing principles and materials, 3D bioprinting can roughly be classified as vat photopolymerization (VP) and ink-based bioprinting [9]. VP, including stereolithography (SLA), digital light processing (DLP), and volumetric printing, is a printing technology that employs UV, visible light, or NIR light to solidify cell-laden photoactive bioresin point-by-point, layer-by-layer, or volumetrically within a vat. Ink-based printing includes extrusion bioprinting and inkjet bioprinting. Extrusion bioprinting constructs 3D structures layer by layer via extruding bioink onto a platform [10]. Inkjet bioprinting uses nozzles similar to those in traditional inkjet printers to deposit tiny biological droplets containing cells and biomaterials layer by layer onto a printing platform to build 3D structures [11]. The viscosity of the bioink for ink-based bioprinting can range from 30 cP to 6 × 10^7^ cP, which means there is a wide range of materials to choose from. The ink-based bioprinter, especially the extrusion-based bioprinter, is cost-effective. Thus, ink-based bioprinting is now the most widely applied method for the fabrication of tissue engineering scaffolds [11,12]. However, the printing resolution of extrusion-based bioprinting is limited to 200–1000 μm, which presents a considerable hurdle when aiming to recreate intricate biological tissues [13]. While the printing resolution of inkjet-based bioprinting is higher, typically ranging from 20–50 μm, it still falls short of the requirements for constructing complex biological tissues [14]. Ink-based bioprinting utilizes nozzles or syringes to extrude bioink, which may lead to low cell viability [15]. In contrast, VP offers unique advantages compared to ink-based bioprinting in terms of manufacturing precision, printing quality, reproducibility, and molding efficiency. The printing resolution for VP-based bioprinting can be less than 100 nm, making VP-based bioprinting a highly promising technology for constructing hierarchical structures that closely mimic natural tissue organization. Thus, VP shows great potential in the customized fabrication of tissue scaffolds, dental materials, soft robots, microfluidics, etc. [16].

In 3D bioprinting, bioink refers to a composite mixture (typically in the form of a hydrogel) composed of one or more types of biomaterials and cells [17]. For VP bioprinting, the term “bioresin” is employed to denote the initial solution that contains cells and from which the 3D objects are polymerized [18]. Over the past decades, substantial strides and achievements have been made in developing bioresin from photoactive biomaterials with the essential biocompatibility and biodegradability to facilitate the creation of functional tissue replicas. A bioresin is composed of photoactive precursors, photoinitiators (PIs), encapsulated cells, and other additives such as growth factors. It must possess exceptional physicochemical and biological properties to ensure both printability and biocompatibility. Biomaterials used in tissue engineering for bioprinting should fulfill several requirements:

1. Printability: The rheology and viscosity properties of bioresin should be compatible with VP to ensure the successful fabrication of intricate and accurate 3D structures with high integrity and high printing precision [19].
2. Biocompatibility: Bioresin before and after photopolymerization should facilitate cell adhesion, proliferation, and differentiation. During the printing process, it is essential that the biomaterials used can protect living cells and other bioactive components from pressure, mechanical forces, and photocrosslinking, as these factors can impact cell fate. The degradation products of the build constructs should also have a minimal impact on cell growth and differentiation [20].
3. Versatility: The inherent characteristics of bioresin, such as adaptability to different printing methods, photocrosslinking mechanisms, and types of incorporated bioactive components, should be adjustable within certain ranges to meet the complex requirements of tissue engineering.

The aim of this review is to give a comprehensive overview of photoactive biomaterials and the current development of VP-based bioprinting, with a specific focus on tissue engineering applications. There are some excellent reviews about light-based bioprinting, but they mainly focus on extrusion-based bioprinting. Our objective is to offer a detailed review of the mechanism of photopolymerization, strategies for VP-based bioprinting, the utilization of photoactive biomaterials for bioresins, and their corresponding applications in replicating various tissues. Moreover, we also aim to provide guidance on fabricating tissue scaffolds using VP-based printing from a materials perspective. In this review, we first introduce the mechanism of photopolymerization. Subsequently, we provide an overview of the VP printing techniques and the commonly used biomaterials for bioresins. The applications of VP-based bioprinting in tissue engineering are then introduced. Finally, perspectives for the future development of VP-based bioprinting are discussed.

## 2. The Mechanism of Photopolymerization

Photopolymerization uses light to initiate the polymerization of photosensitive materials (such as unsaturated acrylate monomers [21], cyclic monomers [22], thiols [23], alkenes [24], etc.). Compared with thermal polymerization, a multitude of substantial benefits emerge from photopolymerization. One of the advantages of photopolymerization is its remarkably rapid reaction rate, with reactions often completing within mere seconds or minutes. Moreover, the process exhibits remarkable efficiency, functioning effectively across a broad spectrum of temperatures, including ambient and even lower temperatures [25]. Moreover, photopolymerization formulations also use minimal quantities of organic solvents, thereby reducing the emission of volatile organic compounds (VOCs). These features endow photopolymerization with characteristics like energy efficiency, adaptability, cost-effectiveness, and environmental friendliness. Now, the applications of photopolymerization have expanded to many fields, such as microfluidics systems [26], microelectronics [27], nanofabrication [28], and 3D and 4D printing [29,30].

**Figure 1:**
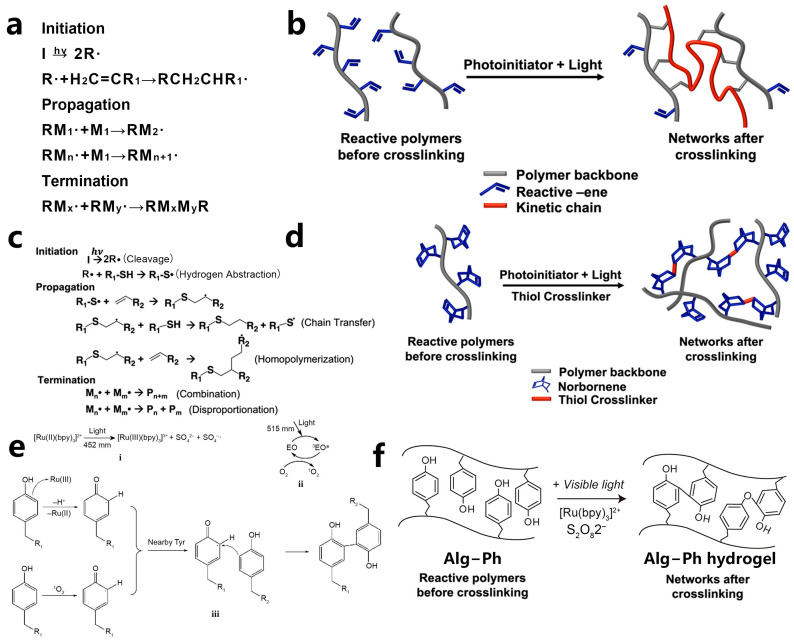
Overview of photopolymerization: (**a**) Illustration of the mechanism for free radical chain growth photopolymerization [36]. Copyright 2020, American Chemical Society. (**b**) Chain growth photopolymerization of monomers or macromers containing unsaturated groups [40]. Copyright 2020, American Chemical Society. (**c**) General mechanism for step growth photopolymerization [36]. Copyright 2020, American Chemical Society. (**d**) Step growth photopolymerization of monomers or macromers containing thiol and norbornene groups [40]. Copyright 2020, American Chemical Society. (**e**) General mechanism for photo radical coupling reaction [41]. Copyright 2021, Elsevier Ltd. (**f**) Photo radical coupling reaction of monomers or macromers containing phenol groups [43]. Copyright 2018, American Chemical Society.

A typical photopolymerization formulation includes photoinitiators (PIs), monomers, and other additives [31]. After exposure to excitation light, PIs absorb energy and generate active fragments, such as free radicals [32], cations [33], or anions [34], which then initiate the polymerization of monomers to further produce polymers. Radicals are more stable in a physiological environment compared to cations and anions. Therefore, free radical photopolymerization finds wide applications in the field of 3D bioprinting. There are primarily four types of photopolymerization techniques utilized in VP-based bioprinting, namely, chain growth photopolymerization [35,36], step growth photopolymerization [36,37], two-photon polymerization [38], and photoradical coupling reactions [39,40,41,42] (Figure 1a–f). This section will introduce these four types of photopolymerization in detail.

### 2.1. Free Radical Chain Growth Photopolymerization

**Figure 2:**
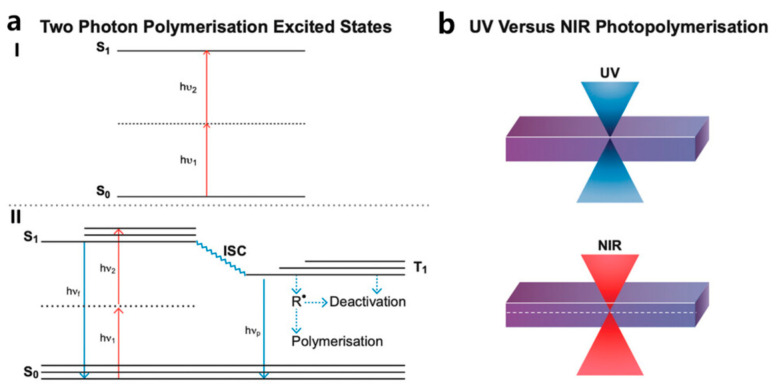
(**a**) The mechanism of TPA. (I) A molecule absorbs two photons and transitions from the ground state to an excited state through simultaneous absorption and a virtual, short-lived intermediate state; (II) The excited molecule either undergoes intersystem crossing (ISC) to generate radicals or returns to the ground state. [83]. Copyright 2022, Wiley-VCH. (**b**) One-photon photopolymerization is characterized by limited light penetration and a pronounced surface interaction with the bioresin, while TPP photopolymerization entails minimal surface interaction and improved laser penetration [83]. Copyright 2022, Wiley-VCH.

In VP-based bioprinting, the free radical chain growth photopolymerization reaction emerges as the most extensively employed technique. It primarily takes three stages: initiation, propagation, and termination (Figure 2a) [40]. It presents several benefits, such as fast speed, high efficiency, a wide range of monomer choices, and ease of implementation. PI is the key component in a photoactive formulation. When irradiated by incident light, the PI undergoes homolysis and generates free radicals to initiate polymerization (Figure 1a,b) [40]. VP-based bioprinting primarily utilizes high-molecular-weight biomaterials that have been modified with acrylate, methacrylate, and methacrylamide groups, for example, gelatin methacrylate (GelMA) [44], hyaluronic acid methacrylate (HAMA) [45], and others [46,47,48]. These materials will be discussed in the material part.

But the photopolymerization of high-molecular-weight biomaterials also faces various challenges, including oxygen inhibition [49], reaction diffusion-controlled kinetics [50,51], incomplete conversion of monomers [52], and heterogeneity within the polymer network [18,53]. Normally, oxygen inhibition can be solved by creating an oxygen isolation environment [54]. However, in bioprinting, oxygen is critical for maintaining living cells’ viability within the crosslinked polymer network. The formed free radicals are easily trapped within the crosslinked network, leading to a high local concentration of radicals. This will result in a significant degree of microstructural heterogeneity in the hydrogel network [55]. Moreover, when the monomers are multifunctional, the free radical photopolymerization product may also be heterogeneous, characterized by extensive crosslinked regions and less densely crosslinked regions [56]. This inhomogeneous network can lead to shrinkage stress within the resulting hydrogel and potentially cause deformation or mechanical failure of the construct, which could in turn impact the fate of cells.

Despite the challenges posed by oxygen inhibition and the heterogeneity of the hydrogel network resulting from chain growth photopolymerization, this non-orthogonal reaction is still preferred in clinical applications because of its simplicity and high efficiency.

### 2.2. Step-Growth Photopolymerization

Step-growth photopolymerization is a kind of photo-click reaction [57]. The merging of photo-click chemistry allows for the creation of a wide range of molecular structures, conjugates, and networks in a spatiotemporally controlled manner in complex systems. These reactions demonstrate broad applicability in constructing 3D hydrogel networks and have gradually evolved into one of the most dynamic research areas for biomedical applications [58,59,60]. Different from free radical chain growth photopolymerization, step growth photopolymerization is a highly selective orthogonal reaction. Typical step-growth photopolymerization includes thiol-ene reactions [61], thiol-epoxy reactions [62], tetrazole-ene cycloaddition [63], and azide-alkyne cycloaddition [64]. Among them, the thiol-ene reaction system has been researched and applied more widely [65].

The mechanism of the thiol-ene reaction is as follows (Figure 1c,d): After irradiation, the radical generated from PI cleavage abstracts a hydrogen atom from the sulfide group in the monomer molecule, leading to the generation of a thiyl radical. This thiyl radical then reacts with the double bond of an alkene molecule, producing a carbon-centered radical. This carbon-centered radical subsequently abstracts a hydrogen atom from another thiol molecule, leading to the polymerization of thiol and alkene monomers [18,40]. Thus, the crosslinking of thiol and alkene mainly follows a step-growth mechanism and polymerizes in a quantitative way. Thus, hydrogels fabricated by step-growth photopolymerization exhibit remarkable uniformity, reduced shrinkage, and decreased mechanical stress, providing a more suitable microenvironment and mechanical cues for cell proliferation and differentiation [66]. Moreover, thiol-ene polymerization proceeds rapidly even in the presence of oxygen. In an oxygenated environment, the carbon-based radical mentioned above can form a peroxyl radical through interaction with an oxygen molecule. The reactivity of this peroxyl radical is high enough to abstract a hydrogen atom from a thiol molecule and thus reinitiate the polymerization process [67,68]. These characteristics make the thiol-ene reaction an optimal approach for constructing hydrogel networks in the presence of living cells. Thus far, a variety of types of cells have been effectively encapsulated within thiol-ene hydrogel networks, for example, human mesenchymal stem cells (MSCs) [69], human umbilical vein endothelial cells (HUVECs) [60], fibroblasts [70], and fibrosarcoma [71].

Despite its numerous advantages, the widespread clinical use of thiol-ene reactions is limited by factors such as high raw material costs and a relatively slower reaction rate when compared to chain growth photopolymerization. Nonetheless, with further research and exploration, this reaction undoubtedly holds vast potential for diverse applications.

### 2.3. Photo-Radical Coupling Reaction

The photo-radical coupling reaction is also used to fabricate hydrogel networks. The monomers used for photo-radical coupling reactions are commonly modified with phenol groups, such as tyramine [72]. Notably, PIs such as dyes or additives are used to initiate the reaction. The PIs undergo a transition to their excited state and then oxidize reactive groups within the reaction system [73]. Eosin Y [74], Bengal Rose [75], and Ruthenium [Ru(II)] [76] are commonly used PIs. The underlying principle varies depending on the specific PI used. In one condition, when [Ru(II)(bpy)_3_]^2+^ is used as a photoinitiator in combination with coinitiator sodium persulfate (SPS), the [Ru(II)(bpy)_3_]^2+^ is oxidized to [Ru(II)(bpy)_3_]^3+^ and subsequently abstracts a hydrogen atom from the phenolic group. This results in the formation of a phenol radical, which subsequently takes part in coupling reactions (Figure 1e). When eosin Y is used as a photoinitiator, it undergoes transitions and intersystem crossings (ISC) to generate triplet eosin Y upon irradiation. Triplet eosin Y can then interact with oxygen molecules through energy transfer to produce singlet oxygen [77]. Subsequently, the singlet oxygen reacts with phenol groups to produce radicals for the coupling reaction [78].

Biocompatible dyes are often used as PIs in this polymerization reaction, and their absorbance wavelength overlaps with visible light. This enables the utilization of visible light as the light source and significantly reduces the issue of cell death caused by radiation [41]. However, the drawback of this method lies in the generation of harmful reactive oxygen species (ROS) during the reaction, which can be detrimental to cells. Therefore, when selecting the PI dosage, it is crucial to keep a balance between polymerization efficiency and the impact of ROS on cells.

### 2.4. Two-Photon Polymerization, TPP

Over the past decade, two-photon polymerization (TPP) has garnered significant attention as an emerging technology to fabricate submicron structures with exceptional spatiotemporal resolution [79,80]. TPP is based on the phenomenon of two-photon absorption (TPA), which was initially proposed by Göppert-Mayer [81]. TPA is a third-order nonlinear optical process. In this process, a photoactive molecule initially absorbs one photon to reach a virtual energy level. Moreover, within a few femtoseconds, it absorbs another photon, leading to the absorption of two photons and transiting from the ground state to an excited state (Figure 2a) [82,83]. Due to experimental limitations, TPA was not observed until 1961, when the observation of TPA was first demonstrated by Kaiser and Garrett at Bell Laboratories [84]. When a femtosecond laser is focused on photoactive bioresin, TPA occurs, leading to the polymerization and solidification of the bioresin [83]. TPA can be realized by utilizing a focused femtosecond laser due to the high pulse peak intensity. Additionally, femtosecond lasers used in TPP have longer wavelengths (normally 600–1000 nm), which results in weaker material absorption but enhanced penetration capabilities. This reduces cell toxicity caused by irradiation and makes in vivo 3D printing feasible. Moreover, polymerization can only occur within the focal plane of the laser beam, as higher energy is required for TPP. This allows for the fabrication of high-resolution freeform 3D structures by simply adjusting the laser’s focal point within the photoactive materials (Figure 2b) [83].

## 3. Vat Photopolymerization (VP)

**Figure 3:**
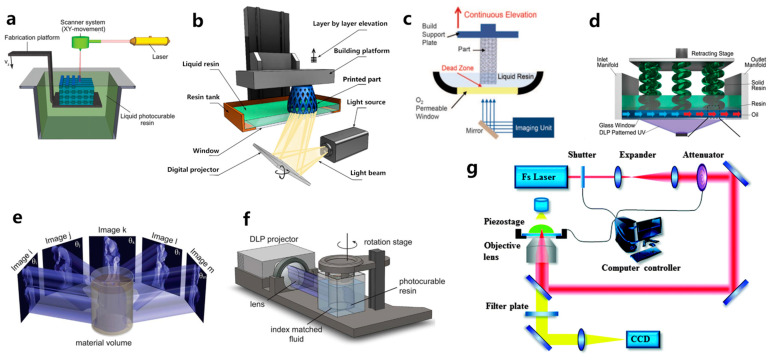
Schematic representation of: (**a**) SLA [88]. Copyright 2017, Wiley-VCH.; (**b**) DLP [89] Copyright 2021, MDPI AG.; (**c**) CLIP [90]. Copyright 2015, AAAS; (**d**) HARP [91]. Copyright 2019, AAAS; (**e**) and (**f**) CAL [92]. Copyright 2019, AAAS; and (**g**) TPP printing [93]. Copyright 2015, The Royal Society of Chemistry.

VP employs light to irradiate and solidify the bioresin within a vat, point by point, layer by layer, or volumetrically (Figure 3a–g). SLA was the earliest VP method and was invented by Charles Hull as an approach to fabricating solid 3D constructs by crosslinking photoactive material layers wisely [85]. Since then, DLP, CLIP, TPP, and CAL have been developed successively. These techniques have received extensive attention due to their exceptional printing precision, rapid production rate, and adaptability in the development of resin materials. By precisely controlling photoactive materials, growth factors, and cells in terms of space and time, VP-based bioprinting can be used to fabricate complex human tissues in vitro, offering great potential for organ and tissue regeneration. Over the past decades, VP has emerged as a dominant tool for creating tissue scaffolds. Currently, extensive research has been conducted on VP for tissue regeneration, encompassing areas like bone [86], skin [16], liver [87], heart [87], blood vessels [8], and so on. In this section, the principles of VP, including SLA, DLP, CLIP, HARP, CAL, and TPP printing, will be introduced.

### 3.1. Stereolithography, SLA

SLA, invented by Charles W. Hull in 1986, is the first commercially available 3D printing technique [94]. For SLA-based bioprinting, it utilizes laser-induced photopolymerization to selectively solidify bioresins point by point to fabricate 3D structures. As shown in Figure 3a, a focused laser scans over the bioresin surface point by point to solidify the bioresin [11]. When a single layer is finished, the printing platform moves downward by a layer in the *Z*-axis direction, and the laser again scans the surface of bioresin to photopolymerize another layer of bioresin. The photopolymerization process is repeated again and again until the 3D constructs are successfully built [88,95]. One of the primary advantages of SLA is its ability to achieve high spatial resolution, thanks to the focused laser beam’s small spot size. Hence, SLA is capable of fabricating intricate 3D structures with high resolution, making it extensively utilized in the fields of tissue engineering. Chan et al. [96] stained NIH/3T3 fibroblast cells with green and red fluorescent dyes, respectively. They then employed a mixture of poly(ethylene glycol) diacrylate (PEGDA) and growth factor RGDS amino acids, which were encapsulated with the stained NIH/3T3 cells for SLA 3D bioprinting. After printing, multilayered wheel-like structures with a layer thickness of 100 μm were fabricated. The printed 3D hydrogel exhibited excellent integrity, high resolution, and high cell viability. After 14 days of in vitro cultivation, noticeable cell migration occurred within the hydrogel containing RGDS growth factors. It indicated the excellent biocompatibility of the SLA-printed hydrogels and the facilitative role of the RGDS amino acid sequence in promoting the movement and migration of NIH/3T3 cells.

Despite its remarkable advantages, SLA also has its limitations. SLA employs lasers as a light source, which makes the 3D printer quite expensive. Furthermore, the bioresin not only needs to be photoactive but also needs to be transparent so that lasers can penetrate the ink and irradiate the photopolymerization of photoactive bioresins. Therefore, the development of cost-effective SLA printing devices and appropriate SLA bioresin materials is the future trend.

### 3.2. Digital Light Processing, DLP

Evolved from SLA, the DLP technique represents the second generation of photopolymerization-based 3D printing. As illustrated in Figure 3b, it differs from SLA by utilizing a patterned exposure technique, enabling the photopolymerization of an entire layer at once [89]. Consequently, even for extremely complex and large structures, the printing time per layer remains constant, which leads to reduced power consumption and higher printing efficiency. This layer-wise solidification approach implies that the more items printed simultaneously, the higher the efficiency gains. Such printing quality and printing speed make DLP technology especially attractive for large-scale industrial production. Compared to other printing methods, DLP offers superior precision, smoother surface quality, enhanced repeatability, improved structural integrity, and better mechanical properties. Moreover, it can also minimize shear forces, which affect the fate of the encapsulated cells [97]. Grigoryan et al. [8] utilized DLP printing to fabricate intricate and functional vascular architectures. PEGDA was used as a monomer, and food dyes were used as PIs. The obtained vascular architectures are capable of exchanging oxygen. This work gained extensive attention when first published, as constructing a complex biomimetic structure that simulates both an intricate vascular system and an airway network poses significant challenges. However, the shortage of photosensitive biomaterials has also emerged as a primary limiting factor in the development of DLP technology.

**Figure 4:**
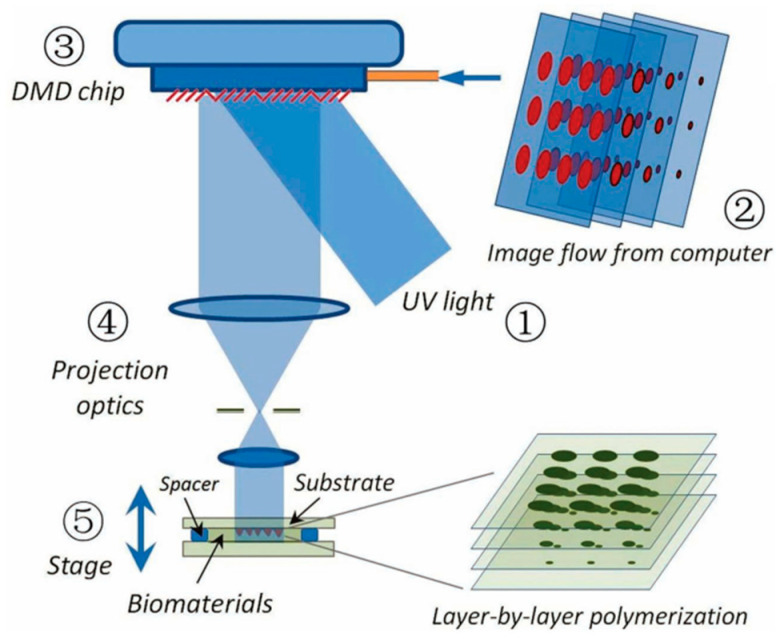
Schematic diagram of DOPsL [98]. Copyright 2012, Wiley-VCH.

Compared to extrusion printing, DLP technology offers significantly enhanced printing resolution. Nevertheless, the layer-by-layer curing process can result in a noticeable staircase effect between adjacent layers, which can lead to low precision along the *z*-axis. To address this issue, Chen’s team [98] introduced a groundbreaking solution known as dynamic optical projection stereolithography (DOPsL) based on DLP. In DOPsL, the printing platform synchronizes its movements with the digital micromirror device (DMD) in the DLP system (Figure 4), enabling continuous high-resolution printing. This approach enables the rapid fabrication of microstructures with smooth sidewalls. By using DOPsL, the research group successfully printed an array of geometric shapes with improved *z*-axis printing accuracy. The geometric shapes included curved micro-hole structures, floral patterns, and spiral formations, all confined within a chip measuring 4.6 mm in length and 3.5 mm in width.

### 3.3. Continuous Liquid Interface Production, CLIP

Compared to SLA, DLP significantly reduces printing time by solidifying an entire layer at once. However, in DLP printing, following the completion of each layer, the printing platform needs to be adjusted—raised or lowered—to allow the refilling of bioresin into the vat. Subsequently, the platform is immersed in the bioresin to continue the printing. This process is repeated again and again until the 3D printing is completed. When printing small-sized structures, the cumulative time taken for platform movement has a minimal impact on efficiency. However, when it comes to the printing of large 3D objects, the increased number of printing layers extends the time needed for the up-and-down motion of the printing platform, thereby significantly decreasing printing efficiency. Consequently, this presents challenges to the practical viability of commercial DLP applications.

To address this challenge, the DeSimone team [90] customized DLP printers and developed the CLIP technique, which enhanced the speed of DLP printing by a factor of 1000. While oxygen inhibition is generally avoided in photopolymerization due to its tendency to cause incomplete photopolymerization and surface tackiness when conducted in the presence of air, DeSimone’s team takes advantage of this effect. As depicted in Figure 3c, the conventional release film at the bottom of the vat of the DLP printer was replaced by an oxygen-permeable polytetrafluoroethylene (PTFE) film. Oxygen permeating from the bottom of the vat inhibits the crosslinking of the bioresin, creating a thin layer of a ‘dead zone’ with a thickness of only several tens of micrometers. Bioresin above this dead zone can still be photopolymerized. This approach blurs the concept of layers and significantly shortens the printing time. The Mirkin team [90] introduced another continuous printing technology called high-area rapid printing (HARP), which involves incorporating a fluorinated oil layer with a thickness of approximately 7 mm between the photoactive resin and the transparent release film. The interface between the resin and the fluorinated oil established a stable solid-liquid boundary, leading to a decrease in adhesive forces between the crosslinked layer and the release film at the bottom. This innovation enables the printing of a 38 cm × 61 cm × 76 cm construct within only 105 min, enabling continuous large-area and rapid vertical printing. The HARP technique is versatile and applicable to various materials. For instance, grid-like structures were printed using polybutadiene rubber elastomer, which can completely regain its original shape after compression.

### 3.4. Computed Axial Lithography, CAL

Despite the rapidity and high precision advantages offered by SLA, DLP, and CLIP, the printing process still follows a layer-by-layer manufacturing approach, which imposes limitations on the achievable speed. Inspired by the principles of computed tomography (CT) imaging, Taylor’s team [55] developed CAL, where arbitrary geometries can be fabricated volumetrically through photopolymerization. As shown in Figure 3e,f, While the photoactive resin container revolves around the vertical axis, a precomputed digital pattern sequence of light is projected into the photoactive resin. As the rotation progresses, numerous diverse projections are emitted into the resin. Over time, the cumulative light exposure solidifies the regions surpassing the photopolymerization threshold while leaving the portions below the threshold unpolymerized. This process enables the creation of the desired 3D structures in a single printing step.

At present, volumetric additive manufacturing has demonstrated its capability to create intricate components at a notable throughput (>105 mm^3^ per hour) and is suitable for a diverse array of materials [99,100,101]. Looking ahead, there is a clear trajectory for the continued advancement of volumetric stereolithography. It is anticipated to encompass several aspects, including enhancements of printing resolution, finetuning of the manufacturing process, optimization of the physical system, and the exploration of its integration across diverse domains.

### 3.5. 3D Printing Based on TPP

**Figure 5:**
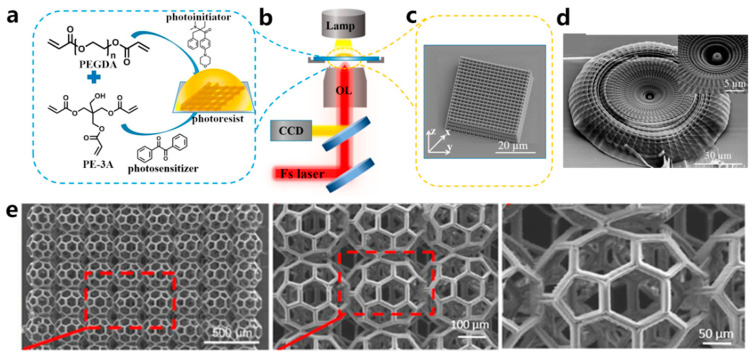
(**a**) The structure of the main components in the bioresin [114]. Copyright 2019, American Chemical Society. (**b**) Illustration of the TPP technique used to fabricate 3D structures [114]. Copyright 2019, American Chemical Society. (**c**) SEM photo of a TPP-printed woodpile structure [114]. Copyright 2019, American Chemical Society. (**d**) SEM photo of a TPP-printed hydrogel scaffold with a straw hat shape. Inset is the magnified photo [114]. Copyright 2019, American Chemical Society. (**e**) SEM images display a scaffold in the shape of a biodegradable buckyball [115]. Copyright 2020, IOP Publishing.

The fabrication resolution of the aforementioned VP methods based on one-photon absorption photopolymerization is limited by the optical diffraction limit. This limitation poses significant challenges in achieving high-resolution 3D structures at the submicron scale [102]. Different from other VP printing methods such as SLA, DLP, CLIP, and CAL, TPP utilizes a near-infrared femtosecond laser, which enables the fabrication of intricate and exceptionally precise 3D microstructures with high resolution, not only at the microscale but also at the nanoscale [93,103,104]. A TPP 3D printer primarily consists of a femtosecond laser pulse system and a platform that can move with sub-micron precision (Figure 3g) [93]. The femtosecond laser is used to generate laser pulses of wavelengths in the range of 600–1000 nm, while the motion platform is responsible for carrying the printing material and 3D focal scanning [83]. The initiation of TPP occurs through third-order nonlinear absorption within the focal area. 3D constructs can be fabricated by guiding the focused laser beam within the photoactive materials along a CAD path, achieving resolution that surpasses the optical diffraction limit. The application of TPP technology has spread across various fields, including microelectromechanical systems (MEMS) [105], microfluidics [106], microoptics [107], biomedicine [108], sensors [109], and microrobotics [110,111]. Since the printing resolution plays a crucial role in replicating the native microenvironment of the ECM for hydrogel constructs, TPP is considered an ideal method for creating tissue scaffolds. Extensive research has been conducted on TPP for hydrogel fabrication [112,113]. Yu et al. [114] prepared a series of scaffolds tailored to different stiffness and configurations using TPP. PEGDA was used as a monomer, and pentaerythritol triacrylate (PE-3A) was used as the crosslinker (Figure 5a,b). By adjusting the concentrations of PEGDA and PE-3A to 40 wt % and 60 wt %, respectively, a fine, intricate 3D structure with a resolution of 80 nm was fabricated (Figure 5c,d). By adjusting the concentration of PE-3A, scaffolds with Young’s modulus ranging from 1.4 to 11.9 MPa were achieved. To improve the biocompatibility of the scaffolds, 2 wt % of chitosan and sodium hyaluronate were incorporated into the PEGDA/PE-3A prepolymer, respectively. Results showed that chitosan improved adhesion of murine fibroblast (L929) cells, while sodium hyaluronate ensured the biocompatibility of the prepolymer.

### 3.6. Light Source for VP

Though TPP is excellent at fabricating complex structures with high resolution, its primary drawback is its comparatively low throughput. To finish the printing within a reasonable timeframe, the TPP-printed structures tend to be rather small. Another limitation is that the biomaterials suitable for TPP are rather inadequate. Thus, Weisgrab et al. [115] modified poly(trimethylene-carbonate) (PTMC) with methacrylic anhydride (MAA) to synthesize the highly photoactive macromer PTMC-MA. Through a systematic study of the printing parameters, they successfully fabricated highly porous scaffolds, PTMAC-MA (96% porosity), with unprecedented large sizes (Figure 5e). After 28 days of cultivation, human adipose-derived mesenchymal stem cells (hADSCs) encapsulated within the porous scaffolds exhibited strong adhesion, proliferation, and differentiation towards their osteogenic and chondrogenic lineages. This work suggests that TPP has the potential to fabricate intricate, large-scale structures. Maibohm et al. [116] proposed a TPP approach that could manufacture large-sized constructs with rapid production and scalability. A fixed diffraction optical element (DOE) was equipped in the traditional TPP apparatus to establish a parallelized, multi-beamlet (nine beamlets) TPP printing process. Using this newly established TPP printing process, they successfully achieved the printing of large periodic 3D microstructure arrays with a high aspect ratio at the sub-micro level, spanning an area of several hundred square micrometers.

As mentioned in previous sections, in VP-based bioprinting, the selection of light sources plays a crucial role in achieving a balance between printing speed and cell viability. The wavelength of the light source should overlap with the absorption spectra of the PIs in the bioresin formulations to initiate the photopolymerization of bioresins. The most frequently used light sources for one photopolymerization fall within the UV and visible light ranges (especially in the UV-A and near-UV visible-light ranges), while TPP-based bioprinting employs NIR laser pulses with wavelengths ranging from 600 to 1000 nm [113,117,118,119]. Thus far, photopolymerization of bioresins has primarily been achieved by employing UV light, as most highly efficient PIs function under UV light [120]. However, UV light may induce genetic mutations and even cause cell death. Consequently, visible light sources are increasingly being employed in VP-based bioprinting to safeguard cell viability and mitigate the potential harm to encapsulated cells [121]. Substituting UV light with visible light not only enhances cell compatibility and expands the application of hydrogel systems, but also provides greater penetration depth, which is beneficial for fabricating hydrogels with more uniform structures [122,123]. Visible light-crosslinked hydrogels have been increasingly employed in diverse fields, including tissue engineering [124], 3D cell encapsulation [125], and drug delivery [126].

TPP-based bioprinting utilizes NIR femtosecond laser pulses, which penetrate more deeply due to the transparency of most bioresins to NIR light. Additionally, NIR light falls within the “biological window”, where light has its maximum depth of penetration in tissue, causing minimal damage to cells and enabling the in vivo 3D printing of cell-encapsulated structures [127]. While NIR femtosecond laser pulses offer deeper penetration for bioresins and provide high manufacturing resolution, a significant limitation of these laser sources is their high cost. Furthermore, the high energy density of the laser device can potentially have a detrimental impact on cell fate [128]. Therefore, it is essential to carefully adjust the energy density to achieve both good fidelity of the printing structure and high cell viability.

### 3.7. Strategies to Improve Printing Resolution

Enhancing the printing speed of VP-based bioprinting is of critical importance for the efficient fabrication of intricate and scalable 3D structures. The speed of photopolymerization is influenced by both the efficiency of PI in the bioresin formulations and the exposure dosage [18]. The exposure dosage is dependent on the irradiation time and the light intensity. One effective approach to increasing printing speed is to reduce the exposure time for each layer, thus reducing the overall printing time. However, this approach may result in lower printing resolution because shorter exposure times can lead to incomplete photopolymerization, further diminishing the fidelity of the printed structure. Another approach to consider is increasing the intensity of irradiation light, which can result in higher crosslinking density and a higher degree of polymerization. However, it is important to note that high-intensity incident light may generate a high density of radicals and lead to difficulties in their diffusion, potentially causing heterogeneity in the polymer network. Moreover, excessive exposure may lead to cell death [120]. Therefore, finding the right balance between cell viability and printing speed is essential for optimizing VP-based bioprinting.

In addition to hardware updates, it is also possible to enhance printing resolution while simultaneously increasing printing speed by fine-tuning the bioresin formulations or developing innovative material toolboxes. In a photopolymerization formulation, PI plays a critical role as it directly influences the photopolymerization kinetics of the bioresin. Choosing PIs with significant absorption overlap with the wavelength of the light source is advantageous for reducing exposure time, thereby facilitating rapid printing, as it allows for the optimization of the solidification time for each layer. Thus far, the most efficient PIs for one-photon polymerization are UV-irradiated and have low water solubility. PIs for two-photon polymerizations are also limited. Thus, developing efficient visible-light PI with good water solubility is urgent. The PIs for VP-based bioprinting are discussed in Section 4.1, Photoinitiators.

Other effective strategies, including changing bioresin solvents [129], designing functional hydrogels [130], and utilizing post-treatment [131], can also enhance the printing speed. Ge et al. [129] employed deep eutectic solvents (DES) as the solvent to dissolve N-hydroxyethyl acrylamide (HEAA) and zwitterionic N-(3-sulfopropyl)-N-(methacryloxyethyl)-N,N-dimethylammonium betaine (DMAPS), creating a mechanically robust photopolymerization ionogel with excellent biocompatibility. This formulation can be used in 3D printing, and the resulting structure exhibited excellent fidelity and high resolution. In VP-based bioprinting, multiple monomers are frequently blended to improve the mechanical properties and biocompatibility of the printed hydrogels. However, the formulation with multiple components might result in poor compatibility among the constituents, potentially leading to reduced printing fidelity. Gong et al. [131] introduced a complexation-induced resolution enhancement 3D printing approach known as “shrinking printing”. Anionic inks, including methacrylated hyaluronic acid (HAMA), GelMA, and chitosan at varying concentrations, were employed as bioinks. After the printing process, the 3D structures were subjected to post-treatment by immersing them in a polycationic chitosan solution. Through charge complexation and the expulsion of water from the hydrogels, the linear dimensions of the hydrogels were reduced to varying degrees.

## 4. Materials for Vat Photopolymerization

A suitable 3D microenvironment is necessary for cell adhesion, proliferation, and migration. Hydrogel is a crosslinked polymer with a 3D network structure, and the hydrophilic functional moieties (–OH, –NH_2_, –COOH, etc.) within the network impart it with tunable water-absorbing capacity, insolubility, and mechanical properties [132]. Natural hydrogels are highly similar to the native ECM and possess hierarchical porous structures, biocompatibility, permeability, flexibility, viscoelasticity, and tissue-like characteristics. Thus, naturally derived biomaterials from organisms, especially those sourced from the ECM, can provide binding sites for cell growth and proliferation, thereby promoting cell differentiation and facilitating the formation of new tissues [133,134]. Natural biomaterials, including collagen, gelatin, chitosan, alginate, and hyaluronic acid, have been widely applied in bioresins due to their excellent biocompatibility, non-immunogenicity, and biodegradability. However, low mechanical performance and poor stability have limited their applications in tissue engineering. In contrast, synthetic hydrogels composed of synthetic polymers often possess better mechanical properties but lower biocompatibility, biodegradability, and functionality compared to natural hydrogels. The most commonly used synthetic hydrogel monomers are polyethylene glycol (PEG), polyacrylic acid, polylactic acid, polyglycolic acid, or poly (lactic-co-glycolic acid) [135,136]. Bioresin composed of natural and synthetic polymers, such as polyethylene glycol-conjugated chitosan and the combination of alginate with methacrylated gelatin (GelMA), are also popular choices for VP-based bioprinting [137,138]. These VP-printed structures not only possess good biocompatibility but also good mechanical properties.

The bioresins employed in VP-based bioprinting are primarily composed of solvent water, photoactive monomers or oligomers, and PIs. The attributes of bioresins, including photoactivity, viscosity, rheology, and functionality, play pivotal roles in printability and fidelity. PI in the bioresin formulation plays a vital role in determining the photoactivity. PI with higher efficiency can help print 3D structures faster and with higher moldability. The viscosity of bioresin used in VP-based bioprinting ranges from 10^−1^ to 10 Pa·s and is normally lower than that of ink-based bioprinting. Lower-viscosity bioresin can reduce printing time and simplify post-processing [139]. Though lower-viscosity bioresin is beneficial for the printing process, it can cause undesired cell sedimentation during long-term bioprinting and result in uneven cell distribution within the printed structures [140]. Therefore, the selection of PI and biomaterials for bioresin formulation is crucial for achieving enhanced printing accuracy and shorter fabrication times.

Over the past decades, many achievements have been made in developing bioresins with biocompatibility and biodegradability to facilitate the fabrication of biologically functional tissue scaffolds. In this section, PIs, natural biomaterials, and synthetic biomaterials used for VP-based bioprinting will be discussed.

### 4.1. Photoinitiators (PIs)

#### 4.1.1. Pis for One Photon Polymerization

The PI used in VP-based bioprinting should possess characteristics such as a high extinction coefficient at the wavelength of the light source, water solubility, and low cytotoxicity to ensure the successful fabrication of 3D objects in the presence of living cells [18]. One-photon PIs can be classified into Norrish Type I and Norrish Type II based on the mechanism by which radicals are generated. Upon irradiation, Type I PIs absorb energy and undergo cleavage, producing two radicals that initiate photopolymerization. While Type II PIs undergo a bimolecular reaction where the excited PI molecules interact with a coinitiator (normally a hydrogen donor) to produce free radicals and further initiate the photopolymerization [36].

**Figure 6:**
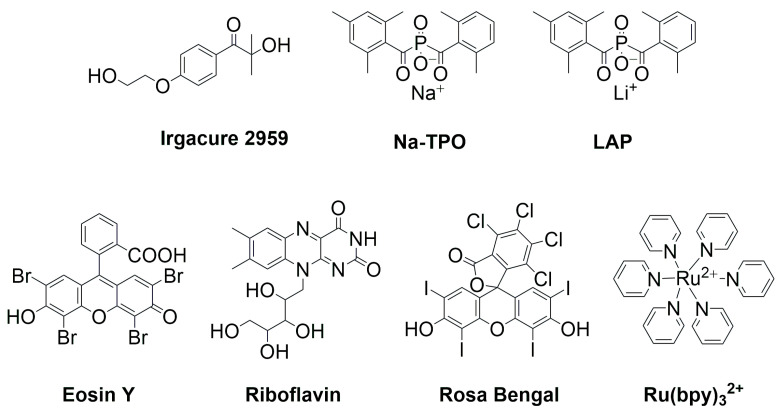
Some of the most commonly used water-soluble one-photon PIs for VP-based bioprinting.

2-hydroxy-1-[4-(2-hydroxyethoxy) phenyl]-2-methyl-1-propanone (Irgacure 2959) is the most commonly used commercial water-soluble Type I PI (Figure 6), and it has been widely applied in bioresin formulations due to its good cytocompatibility [141]. However, the solubility of Irgacure 2959 in water is quite limited, and its absorption falls into the UV range, which has significantly hindered its application in cell-encapsulated VP-based bioprinting. Recently, many commercial printers have started to use 405 nm LEDs as their light source. Consequently, there is a growing research demand for developing high-efficiency, water-soluble, visible-light-sensitive PIs with good biocompatibility. PPhosphinoxides are a class of highly efficient Type I photoinitiators that absorb light with broad absorption in the UV-Vis range [142,143]. Monoacylphosphineoxide (MAPO) and bisacylphosphineoxide (BAPO) salts, including lithium phenyl-2,4,6-trimethylbenzoylphosphinate (LAP) and sodium phenyl-2,4,6-trimethylbenzoylphosphinate (Na-TPO) (Figure 6), are highly reactive and biocompatible visible light PIs with absorption ranges from 380 nm to 450 nm [144]. Significantly, these PIs also boast excellent water solubility and much lower toxicity than Irgacure 2959 [145].

In addition to traditional PIs, dyes such as riboflavin [146], eosin Y [147,148], and Rosa Bengal [149] with good biocompatibility have recently been utilized in bioresin formulations (Figure 6) [150]. Riboflavin, more commonly known as vitamin B, is a Norrish type II PI that exhibits exceptional water solubility and biocompatibility [151,152]. The absorption spectrum of riboflavin spans from 300 to 500 nm, making it an ideal PI for visible light-induced photopolymerization [153]. Eosin Y, another dye often used as a histological stain, was recently applied as a PI for VP-based bioprinting [154,155]. Its absorption peak is at 528 nm, and it exhibits enhanced photoinitiation efficiency when used in conjunction with ethyl-4-dimethylaminobenzoate (EDAB). Rose Bengal has emerged as another candidate for Type II PIs, with its absorbance occurring at 565 nm [156,157]. Another noteworthy water-soluble visible light PI is Tris(2,2′-bipyridyl)dichloro-ruthenium(II), or Ru(bpy)_3_^2+^. This metal complex-derived compound exhibits a pronounced absorption peak at 452 nm [158]. Upon exposure to visible light, Ru^2+^ undergoes a transition to its excited state and interacts with SPS through an electron transfer process to generate Ru^3+^ and sulfate radicals. The sulfate radicals subsequently initiate the photopolymerization of monomers [159].

#### 4.1.2. PIs for TPP

In contrast to single-photon absorption, 2PIs absorb two photons simultaneously, which allows the use of near-infrared (NIR) light. The advantages of NIR light are improved depth penetration in tissue and less harm to living cells compared to UV light.

**Figure 7:**
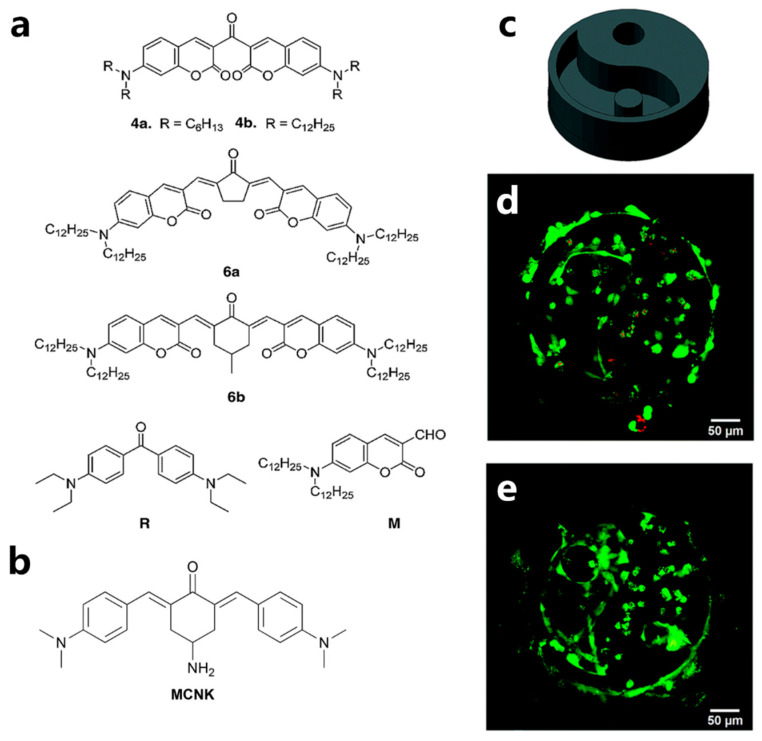
(**a**) Chemical structure of a group of innovated TPA PIs and reference PIs [163]. Copyright 2014, American Chemical Society. (**b**) Structure of MCNK [164]. Copyright 2017, The Royal Society of Chemistry. (**c**) A digital model of Ying-yang, which is used for TPP printing [164]. Copyright 2017, The Royal Society of Chemistry. (**d**) After 24 h of cultivation, the loaded MC3T3 cells with a round morphology indicated their adhesion and stretching [164]. Copyright 2017, The Royal Society of Chemistry. (**e**) After 5 days of cultivation, the encapsulated cells showed good viability [164]. Copyright 2017, The Royal Society of Chemistry.

Traditional PIs used for one-photon polymerization have smaller TPA cross-sections (σ_TPA_), resulting in lower photoinitiation efficiency for TPP and requiring higher laser energy for the initiation of polymerization [160,161]. However, excessive laser energy can lead to optical damage to the bioresin, microstructures, and encapsulated cells. Highly active TPP PIs can effectively increase processing speed and reduce the laser energy needed. Therefore, the development of novel and efficient TPP PIs is of crucial importance. The key factor in designing TPP PIs is to create molecules with large σ_TPA_. σ_TPA_ is proportional to the intramolecular charge transfer (ITC) amount, and various approaches, such as extending the conjugation chain length, introducing strong electron-donating or electron-withdrawing groups onto the molecule, and increasing molecular dimensions, can all increase the ITC amount, thereby enhancing the σ_TPA_ of the molecules [162]. Nazir et al. [163] developed a series of biocompatible π-extended coumarins with high σ_TPA_ values (maximum σ_TPA_ values around 400 GM) by modifying coumarin with different dialkylamino groups (Figure 7a). The synthesized PIs exhibited excellent photoinitiation efficiency and biocompatibility. MC3T3-E1 preosteoblastic cells cultured on the TPP printing films displayed spindle-shaped morphology after 7 days of cultivation. Furthermore, improved proliferation of MC3T3-E1 was also observed. The results indicated that the synthesized PIs possess excellent biocompatibility. However, it is worth noting that the migration of the small molecular PIs to the ECM surface may potentially lead to toxicity in other parts of the human body. To address this issue, Tromayer et al. [164] synthesized a highly efficient water-soluble macromolecular TPP PI based on benzylidene ketone dyes. The synthesized PI exhibited significant cytotoxicity and phototoxicity. They then combine amino-cyclohexanone TPP PI (MCNK, Figure 7b) with a polymeric hyaluronan backbone (HAPI). The resulting HAPI displayed excellent photoinitiation efficiency and significantly improved cytocompatibility. Using HAPI as PI, the TPP printing speed of up to 100 mm/s was achieved, enabling the production of intricate 3D hydrogel structures based on gelatin. The encapsulated MC3T3 cells exhibited a round morphology after 5 days of cultivation (Figure 7c,d). These results indicate that HAPI PI has substantial potential for fabricating cell-laden hydrogels with complex structures.

### 4.2. Natural Biomaterials

#### 4.2.1. Collagen and Gelatin

**Figure 8:**
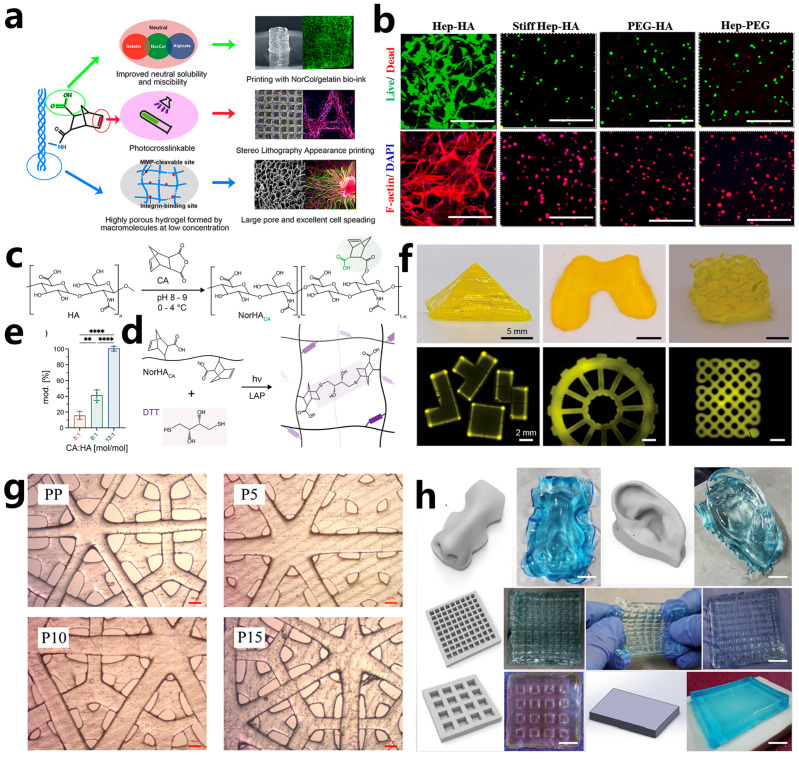
(**a**) The structure of NorCol and its molecular design for enhancing printability and bioactivity, and the morphology of the encapsulated cells in different hydrogels after cultivation [168]. Copyright 2021, American Chemical Society. (**b**) Fluorescence images of ADSCs after 21 days of cultivation in different hydrogel scaffolds (scale bar: 300 μm) [190]. Copyright 2017, Elsevier Ltd. (**c**) Synthetic routine of NorHA macromers and hydrogels. (**d**) schematic illustration of the photopolymerization of NorHA/DTT [191]. Copyright 2023, American Chemical Society. (**e**) The change in HA modification degree with the change in molar ratio of CA to HA [191]. Copyright 2023, American Chemical Society. Significance determined at *p* < 0.05, ** *p* < 0.01; **** *p* < 0.0001. (**f**) Images of 3D printed pyramid, femoral condyle, and 3D gyroid (scale bar: 5 mm) and fluorescence photos of 3D building blocks, wheel, and knotted pattern (scale bar: 2 mm) printed by using NorHA bioresin [191]. Copyright 2023, American Chemical Society. (**g**) Optical microscopic photos of DLP-fabricated three-layer hydrogel with different bioresin compositions [197]. Copyright 2017, Elsevier Ltd. (**h**) DLP-printed CHMA/PAN hydrogels with different structures (scale bar: 10 mm) [203]. Copyright 2021, Elsevier Ltd.

Collagen, a protein of substantial functional importance, is abundantly present in mammalian organisms and serves as a fundamental building block of muscles and bones [165]. Featuring a triple helix structure, collagen molecules can autonomously assemble into gels, offering mechanical strength, viscoelasticity, and bioactive sites essential for human tissues. Gelatin, derived from denatured collagen, contains the arginine-glycine-aspartic acid (RGD) peptide sequence, enabling cell attachment and spreading across the hydrogel matrix. Notably, both collagen and gelatin molecules possess excellent biodegradability and biocompatibility, which are beneficial for cell adhesion, proliferation, and migration. Hence, collagen and gelatin hold substantial promise in the fields of tissue engineering and cellular behavior investigation [166]. By modifying collagen and gelatin with methacrylic anhydride (MAA), methacrylated collagen (ColMA) and gelatin (GelMA) with photoactivity can be synthesized, respectively. Noohi et al. [167] isolated type I collagen from three distinct animal sources: bovine Achilles tendon, bighead carp fish skin, and rat tail tendons. Furthermore, they modified these collagens with MAA to fabricate ColMA. Subsequently, the three kinds of ColMA were separately mixed with PEGDA and irradiated by light to fabricate 3D photocrosslinked networks for the cultivation of human corneal stromal cells (hCSCs). They observed that the hydrogel containing rat tail type I collagen exhibited larger pore diameters and higher porosity, which facilitates the penetration of hCSCs. In contrast, the hydrogel containing fish skin collagen showed higher expression of type I collagen and lumican. These findings emphasize how the source of collagen extraction significantly influences cellular behavior and highlight the importance of making a suitable choice to match the intended application of the tissue-engineered hydrogel. Guo et al. [168] modified collagen with itaconic anhydride to prepare norbornene-functionalized neutral soluble collagen (Nor-Col), which is suitable for thiol-ene photo-click reactions (Figure 8a). Nor-Col molecules contain numerous carboxyl groups, enhancing their compatibility with biopolymers like gelatin and sodium alginate. Consequently, these three components can be directly mixed and utilized as bioresin for SLA. Assisted by a thiol cross-linker, this three-component bioresin exhibited exceptional printability and fidelity. Moreover, after 14 days of cultivation, rapid spreading and robust connections of human dermal fibroblasts (HDFBs) within Nor-Col hydrogels were observed, attributed to the porous structure of the hydrogel (Figure 8a).

GelMA possesses various characteristics, including biocompatibility, enzymatic degradation, cell adhesion, and tunable mechanical properties, making it an excellent candidate for fabricating tissue engineering scaffolds [169,170,171,172]. However, the photocrosslinked network of GelMA may exhibit heterogeneity, which can be detrimental to the cell fate. To address this issue, gelatin derivatives suitable for thiol-ene photopolymerization, such as norbornene-functionalized gelatin (GelNB) [173,174], allylated gelatin [175], and thiol-functionalized gelatin (GelSH) [176], have been synthesized. Munoz et al. [35] conducted an experiment in which they fabricated 3D constructs by the photocrosslinking of GelNB and a bifunctional crosslinker, dithiothreitol (DTT), at varying concentrations under UV irradiation. Their study revealed that the human mesenchymal stem cells (hMSCs) encapsulated in GelNB/DTT hydrogels exhibited higher cytocompatibility compared to those in GelMA hydrogels. Additionally, the researchers also used a tetrafunctional thiol crosslinker, four-armed thiol-terminated polyethylene glycol (PEG4SH), for comparison. When the concentration of GelNB and the ratio between the alkene and thiol groups were held constant, the PEG4SH/GelNB hydrogel exhibited an equilibrium shear modulus of 5 kPa, while the DTT/GelNB hydrogel displayed a shear modulus of only 0.4 kPa. This indicates that functionality also influences the efficiency of step-growth photopolymerization.

#### 4.2.2. Hyaluronic Acid (HA)

HA is a non-sulfated glycosaminoglycan consisting of disaccharide units composed of N-acetyl-D-glucosamine and β-D-glucuronic acid linked by β-1,3 and β-1,4 glycosidic bonds. It is widely distributed in the connective, epithelial, and neural tissues of mammals and possesses excellent properties, including viscoelasticity, biocompatibility, bioresponsiveness, non-immunogenicity, non-coagulability, biodegradability, and hydrophilicity [41]. Due to its remarkable hydrophilicity, HA can modulate the viscoelastic properties of biological fluids by forming non-covalent interactions with water molecules [177]. As a result, it plays a crucial role in cell migration, proliferation, differentiation, osteogenic induction, angiogenesis-promoting properties, cellular mineralization, and vascular regeneration [178,179,180]. Moreover, HA possesses several cell binding motifs, including CD44 (a receptor for MSCs), hyaluronan-mediated motility receptor (RHAMM), and intercellular cell adhesion molecule-1 (ICAM-1), making it an attractive material for promoting cell adhesion and proliferation [181]. Due to their unique biological and physicochemical properties, as well as their safety, natural HA and its derivatives have become intriguing biomaterials in various fields, including medicine [182], pharmaceuticals [183], plastic surgery [184], and others. Among these, hydrogels based on HA have been extensively applied in various fields, including drug delivery [185,186], wound dressings [187], and tissue engineering [179,188]. As a large amount of hydroxyl and carboxyl groups are present in its molecules, HA can be modified by incorporating photoactive groups such as methacrylate, vinyl, thiol, norbornene, and tyramine functional groups into its backbone via crosslinking, grafting, and esterification reactions [189].

Gwon et al. [190] developed a stem cell-responsive hydrogel through visible-light thiol-ene photopolymerization. They used heparin-hyaluronic acid (Hep-HA) and methacrylated hyaluronic acid (HAMA) as monomers, along with eosin Y as the PI. The Hep-HA hydrogel could be tailored to different mechanical properties by adjusting parameters such as light intensity, PI concentrations, raw material concentrations, or swelling ratio. Heparin-PEG (Hep-PEG) and PEG-HA hydrogel were used as controls for comparison. After swelling in PBS, the storage moduli of 2 wt % and 5 wt % Hep-HA hydrogel were 380 ± 50 Pa and 3090 ± 320 Pa, respectively. ADSCs showed good adhesion and proliferation ability on both 2D Hep-HA and Hep-PEG hydrogel surfaces, while they did not adhere and proliferate well on PEG-HA hydrogel surfaces. When ADSCs were cultured in a 3D hydrogel network, it was found that only Hep-HA hydrogel provided good support for the spreading, proliferation, and migration of ADSCs (Figure 8b). These results indicate the importance of heparin moieties in providing binding sites for ADSC adhesion, thus enhancing the biocompatibility of the synthesized hydrogel. Moreover, ADSCs were also seeded on hydrogels with different stiffnesses. The results demonstrated that ADSCs displayed improved adhesion and proliferation on Hep-HA films with lower stiffness. This further indicates that mechanical properties play a significant role in cellular activities.

Galarraga et al. [191] synthesized norbornene-modified HA (NorHA) suitable for thiol-ene photoclick reactions through the esterification reaction of HA with carboxylic anhydride in an aqueous solution (Figure 8c). Upon irradiation, NorHA and DTT can form hydrolytically unstable networks because the carboxylic groups present in the NorHA backbone can accelerate the degradation of the formed hydrogel (Figure 8d,e). By varying the macromer concentration and the degree of HA modification, a range of compressive moduli for NorHA hydrogels from 1.5 to 150 kPa and degradation times spanning from 3 to 14 days can be obtained. DLP was utilized to produce a variety of hydrogels with intricate and macroporous 3D structures, as shown in Figure 8f. These hydrogels demonstrated a strong capacity to facilitate cell attachment.

#### 4.2.3. Chitosan

Chitosan is a product obtained by deacetylation of chitin, primarily composed of amino-glucosamine units. It exhibits a range of functions as a polysaccharide macromolecule, including biodegradability, biocompatibility, non-toxicity, and antimicrobial properties [192]. Its structural units are similar to those found in natural substances like hyaluronic acid and glycosaminoglycans, which is why chitosan finds wide application in various biomedical fields such as drug delivery materials [193], absorbable medical materials [194], tissue engineering scaffolds [195], and pharmaceutical development [196]. Cheng et al. [197] incorporated varying concentrations of chitosan with a 95% deacetylation degree into a solution of poly(ε-caprolactone)-diacrylate/PEGDA to prepare a photoactive bioresin. They observed that with an increase in chitosan concentration, both the tensile strength and degradation time of the photopolymerized hydrogel decreased, while the hydrophilicity of the prepared hydrogel increased. The bioresin, which contained 0%, 5%, 10%, and 15% chitosan, respectively, was DLP printed, resulting in multi-layer scaffolds as shown in Figure 8g. The bioresin showed good printability and fidelity for printing the alternative stacking patterns. After 5 days of in vitro cultivation, L929 fibroblasts seeded on the hydrogel containing 15% chitosan were evenly distributed across the surface, and the presence of microfilament bundles was observed. This demonstrates that chitosan can enhance cell adhesion and proliferation.

The abundance of amino groups present in the repeating units of chitosan offers numerous modification sites for grafting, enabling the fabrication of diverse types of hydrogels based on chitosan derivatives [198,199,200]. Chitosan is commonly modified through acylation, esterification, N-alkylation, and other methods [198]. A common modification method involves the reaction of glycidyl methacrylate with the amino groups on chitosan to achieve methacrylation of chitosan (CSMA). Moreover, thiolated chitosan (CSSH) is also widely used as a tissue engineering material. It possesses high mucoadhesive properties due to the formation of disulfide bonds with mucus glycoproteins on mucosal surfaces, leading to in situ gelation resulting from the formation of inter- and intra-chain disulfide bonds [201,202]. However, chitosan also has some significant drawbacks that hinder its development in the field of VP-based bioprinting. Chitosan molecules contain both amino and hydroxyl groups, which can form intra- and intermolecular hydrogen bonds, significantly influencing the solubility, viscosity, and crystallinity of chitosan. Moreover, chitosan itself does not possess photoresponsiveness, which limits its application in VP-based bioprinting. He et al. [203] proposed a hybrid bioresin that combines methacryloyl chitosan (CHMA), acrylamide (AM), and LAP for DLP-based 3D bioprinting. The compressive strength can be improved from 97 kPa to 345 kPa by increasing the mass ratios of CHMA and AM from 1:5 to 1:20. Moreover, by adjusting the mass ratio of CHMA and AM, a balance between biological and mechanical properties can be achieved. The DLP-printed 3D structures also showed good structural integrity and excellent biocompatibility (Figure 8h). In another work from the same group, CHMA aqueous solution was used as bioresin for DLP bioprinting. They successfully increased the CHMA water solubility from 7.4 mg/mL to 32.0 g/mL by increasing the degree of substitution (DS) of chitosan from 11.7% to 33.6%. With the increased solubility of chitosan, a 1 wt % CHMA solution encapsulated with human umbilical vein endothelial cells (HUVECs) was used as bioresin for DLP, which showed good printability. The viability of HUVECs remained high after 2 days of cultivation. This indicated that photoactive chitosan derivatives have the potential to be used as VP bioresins.

#### 4.2.4. Sodium Alginate

Sodium alginate is a natural polyanionic polysaccharide composed of 1,4-linked β-D-mannuronic acid (M block) and 1,4-linked α-L-guluronic acid (G block) units, often extracted from various algae species in nature [204]. The presence of M units imparts biocompatibility to the molecule, while the mechanical properties depend on the quantity of G units in the molecule [205,206]. Alginate possesses several advantages, including good biocompatibility, low toxicity, immunogenicity, and flexibility. It finds widespread applications in areas such as wound dressings [207], tissue engineering [208], and drug delivery [209]. Yang et al. [210] designed a two-step synthesis method for the fabrication of high-performance alginate/polyacrylamide hydrogels. Firstly, they prepared sodium alginate/polyacrylamide hydrogels and then immersed them in solutions containing divalent or trivalent cations. By controlling the ion exchange process, ionically corosslinked alginate hydrogels with high strength and toughness can be achieved. The results demonstrated that alginate hydrogels crosslinked with trivalent cations exhibited significantly higher strength and modulus compared to those crosslinked with divalent cations.

However, conventional ionically crosslinked alginate hydrogels tend to lose their mechanical strength in in vitro culture, and sodium alginate lacks cell adhesion sites. Moreover, pure ionically crosslinked sodium alginate hydrogels often require higher concentrations to enhance the integrity of the printed 3D structures, while a high concentration of bioresin may increase the stress on cells. By reacting the carboxyl groups of sodium alginate with glycidyl methacrylate (GMA), 2-aminoethyl methacrylate (AEMA), or MAA, photoactive methacrylated sodium alginate suitable for VP bioprinting can be obtained, thus more stable 3D structures can be printed at lower alginate concentrations, which is more conducive to cell growth [189,211].

### 4.3. Synthetic Biomaterials

Compared to natural biomaterials, synthetic biomaterials possess higher mechanical strength, greater flexibility, and ease of processing. Importantly, they can be non-toxic and biodegradable. Their drawbacks lie in the lack of cell recognition sites and cell adhesion ability, resulting in relatively lower biocompatibility when compared to natural biomaterials. The most frequently employed synthetic biomaterials in tissue engineering include polyethylene glycol (PEG) and its derivatives. PEG exhibits amphiphilic properties, extremely low immunogenicity, and high biocompatibility, making it an excellent candidate for tissue engineering [212]. The chain length of PEG can be adjusted to impart PEG-based hydrogels with varying viscosity, rheology, and stiffness [213,214]. Furthermore, through the functionalization of PEG with various moieties such as acrylic acid, methacrylic acid, ethylene, and allyl, it is possible to obtain PEG diacrylate (PEGDA), PEG dimethacrylate (PEGDMA), and multi-armed PEGs [215,216]. Many of these derivatives are photoactive, which makes them quite popular in VP-based bioprinting.

In addition, PEG is inherently nonadhesive to cells and various types of proteins [217]. Leveraging this property, it is possible to build blank blocks loaded with various growth factors and cells, facilitating research into cell adhesion, proliferation, and differentiation. Qu et al. [217] employed SLA to fabricate PEGDA hydrogels patterned with stripe-like and stepped structures bearing RGD peptide. They investigated the impact of scaffold structure and RGD peptide on the proliferation and differentiation of hADSCs. The results revealed that ADSCs tend to grow along the gel walls in the stripe-like PEG hydrogel building block, while they prefer random growth in the stepped-structured PEG hydrogel building block.

## 5. VP in Tissue Engineering

The tissues and organs of the human body exhibit varying levels of complexity. The microstructure of printed scaffolds plays a crucial role in guiding cellular behavior and enhancing cell adhesion, proliferation, and migration. Different cell types have shown diverse responses to various ECMs characterized by differing compositions, microstructures, and surface properties [218]. Evidence indicates that replicating the hierarchical porous structures found in human tissues holds significant importance [219]. However, simulating such intricate tissue structures poses a significant challenge in replicating the complex tissue microenvironment. The advent of 3D bioprinting, particularly VP-based bioprinting, has enabled the creation of intricate microstructures that closely replicate the microenvironment of real human tissues. Thus far, VP-based bioprinting has been used to fabricate tissues such as liver [220], skin [221], bone and cartilage [222,223], and blood vessels [224]. These applications will be introduced in this part.

### 5.1. Liver

**Figure 9:**
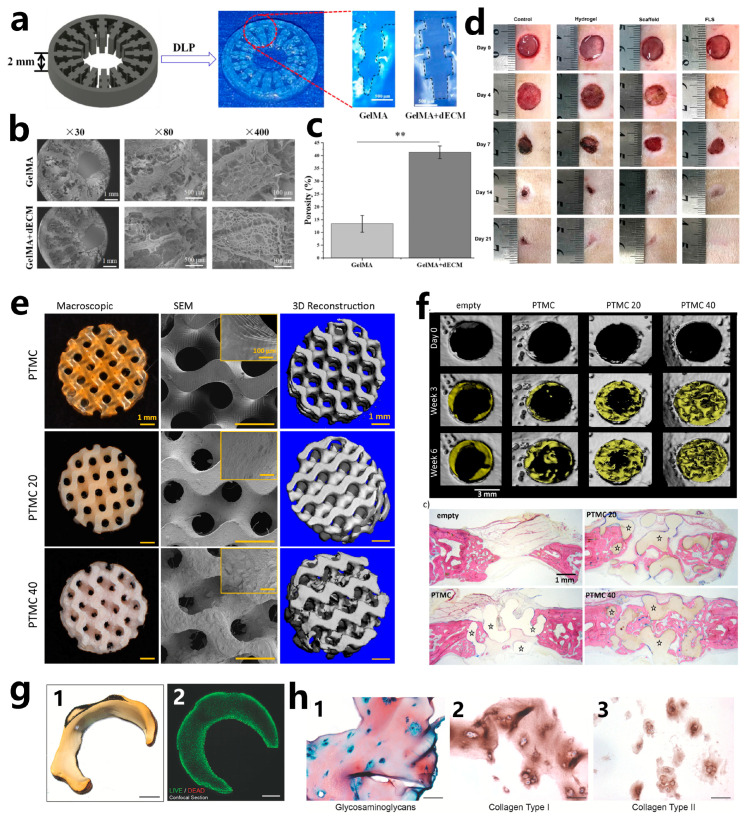
(**a**) The designed digital model of liver microtissue and the corresponding structure printed by DLP. By adding dECM to the bioresin, better printability is achieved [227]. Copyright 2020, Elsevier Ltd. (**b**) The printed liver microtissues show porous structures. Copyright 2020, Elsevier Ltd. (**c**) The porosity of bioprinted liver microtissues [227]. Significance determined at *p* < 0.05, ** *p* ≤ 0.01. Copyright 2020, Elsevier Ltd. (**d**) FLS for full-thickness skin defect repair in a rat model [231]. Copyright 2020, Elsevier Ltd. (**e**) Macroscopic, microscopic, and CT monitoring images of different scaffolds [236]. Copyright 2017, Elsevier Ltd. (**f**) The stimulation of PTMC/HA in vivo mineralization and bone formation at 0, 3, and 6 weeks, respectively. Newly formed bone tissue can be seen from the Giemsa-Eosin staining images [236]. Copyright 2017, Elsevier Ltd. (**g**) The human-sized meniscus structure printed by CAL (1) after 28 days of in vitro cultivation, scale bars: 2 mm, and (2) high cell viability within the meniscus hydrogel cultured for 7 days, scale bar: 2 mm [243]. Copyright 2019, Wiley-VCH. (**h**) After 28 days of cultivation, matrix components are present in the meniscus: (1) GAGs, (2) extensive amounts of collagen type I, and (3) lower amounts of collagen type II (scale bars: 50 μm) [243]. Copyright 2019, Wiley-VCH.

The liver, the human body’s largest gland, plays a crucial role in metabolism, bile production, detoxification, and the regulation of water and electrolytes. Over the past few decades, liver disease has become one of the leading causes of human death worldwide. It is urgent to develop liver scaffolds and in-vitro liver models for liver regeneration, drug screening, metabolism, and hepatotoxicity studies [225]. VP bioprinting has been utilized for the biofabrication of liver tissue [226]. Mao et al. [227] developed liver-specific bioresins by combining GelMA with liver decellularized extracellular matrix (dECM), and human-induced hepatocytes (hiHep cells) were encapsulated to form cell-laden bioresins. An innovative liver microtissue with an inner gear-like structure has been developed to enhance hepatic functional restoration (Figure 9a). The liver microtissue was fabricated using DLP printing. By adding liver dECM to the bioresin, the printability, the porosity of the microtissue, and the viability of encapsulated hiHep cells all increased (Figure 9b,c). When dECM was used, hiHep cells spread within the printed liver microtissue structure further, and hepatocyte-specific functions such as albumin secretion and urea production were enhanced. The results suggest that using liver dECM as a bioresin for cell-encapsulated bioprinting holds significant promise for the fabrication of liver microtissues. Ma et al. [228] utilized DLP to fabricate a hexagonal GelMA/HAMA hydrogel hepatic model tailored with stiffness similar to that of the liver. The human-induced pluripotent stem cells (hiPSCs), along with support HUVECs and adipose-derived stem cells (ADSCs), were encapsulated in the liver model. A more pronounced development of spheroids compared to the model consisting only of hiPSC-HPCs in the 3D triculture model was observed after 7 days of cultivation. These approaches could potentially serve as valuable products in liver tissue engineering for the restoration of hepatic functions.

In recent years, significant progress has been made in the field of 3D bioprinting liver tissue. However, the fabrication of in vitro 3D liver tissue that accurately mimics the biochemical and physiological features of natural liver tissue remains challenging. The construction of a high-density biomimetic vascular network represents one of the bottlenecks in liver tissue biofabrication. Although some studies have utilized multi-material printing techniques to achieve the printing and culturing of endothelial cells and liver cells simultaneously, the formation of perfusable, high-density vascular networks remains difficult.

### 5.2. Skin

Skin, the body’s largest organ, possesses an intricate structure and consists of three layers: the epidermis, the dermis, and the subcutaneous tissue [229]. However, the repair and regeneration of large-scale skin injuries pose significant clinical challenges. The emergence of 3D bioprinting technology offers a new approach to fabricating biologically functional skin graft substitutes. Borris et al. [230] utilized biomaterial-based strategies and bioengineering methods to create a 3D skin model that mimics the multilayered structure of natural skin. This model incorporates layers of endothelial cell networks, dermal fibroblasts, and multiple layers of keratinocytes. The examination of the mechanical characteristics of GelMA-based bioresins blended with varying ratios of alginate revealed that the bioprinted endothelial layer could be more effectively simulated for the enhancement of endothelial cell viability when employing a combination consisting of 7.5% GelMA and 2% alginate. They also found that the stiffness of the hydrogel played a vital role in the regulation of Pro-Collagen I alpha-1 and matrix metalloproteinase-1 production in human dermal fibroblasts. Furthermore, the repeated gelatin coating of human keratinocytes has been proven advantageous for reducing culture time while preserving their viability, enabling the creation of multiple layers of keratinocytes. Zhou et al. [231] proposed a DLP bioprinted multilayer functional living skin (FLS) using a biomimetic bioresin composed of GelMA, N-(2-aminoethyl)-4-(4-(hydroxymethyl)-2-methoxy-5-nitrosophenoxy) butanamide (NB), linked hyaluronic acid (HA-NB), and LAP. The FLS exhibited interconnected microchannels that help enhance cell migration, proliferation, and the formation of new tissue. The bioresin exhibited fast gelation kinetics, adjustable mechanical characteristics, excellent biocompatibility, and effective tissue adhesion. DLP-based bioprinting technology offers a swift approach for accurate placement of clusters of human skin fibroblasts (HSFs) and HUVECs, while a notable level of cell viability can be achieved. Therefore, FLS can be fabricated successfully. A skin defect model in rats was displayed to evaluate the therapeutic efficiency of the fabricated FLS. Results showed that the wound closure time for FLS was significantly shorter than the control group (Figure 9d). These new bioresins and 3D skin substitutes showed great potential for skin regeneration.

The advancements of bioprinting technology offer the possibility of manufacturing more complex skin tissue structures. However, achieving bioprinting of fully functional skin tissue still presents certain challenges. The epidermis and dermis of the skin contain different cell populations, ECM compositions, appendages, and blood vessels. Among these, the formation of vascular networks and the regeneration of skin appendages are the primary challenges in the current bioprinting of skin tissue. To address these challenges, the development of skin-specific biomimetic bioresins is a crucial area of development.

### 5.3. Bone and Cartilage

Bone is a vital tissue in the human body, providing support and protection for human organs. The self-repair capacity of human bone tissue is limited and cannot facilitate the self-healing of large bone defects. Bone transplantation surgery is currently the main method for bone tissue repair and reconstruction. However, bone transplantation surgery faces challenges such as a shortage of donors and issues related to immune rejection. Bone tissue engineering has become an ideal choice [232]. An optimal bone tissue engineering scaffold should meet the following criteria: (1) possessing mechanical properties akin to native bones to provide sufficient support for the human body; (2) demonstrating osteoconductive and osteoinductive properties to facilitate the cells differentiation into osteogenic cells; and (3) integrating with blood vessels to enhance oxygen and nutrient transportation, thereby enhancing cell viability, migration, and proliferation [233,234]. Utilizing VP-based bioprinting to fabricate bone structures ensures the uniform distribution of bone-related cells, reduces the occurrence of necrotic areas, and enables the integration of blood vessel structures. Kufelt et al. [235] synthesized glycidyl methacrylated HA (HAGM) suitable for TPP by treating HA with glycidyl methacryla (GMA). HAGM/PEGDA hydrogels were prepared through photopolymerization. By adjusting the content of PEGDA, the hydrogels can be tailored to a variety of storage moduli ranging from 0.03 to 0.3 MPa, which is similar to that of soft rubber. Furthermore, to enhance the bioactivity of HAGM even further, epidermal growth factor (EGF), which plays a critical role in cell activity, was incorporated into HAGM by using N-succinimidyl acrylate linkage. The growth potential of bone increased up to 177%, compared to 68% for the hydrogel without incorporating EGF. Guillaume et al. [236] synthesized photocrosslinkable methacrylated poly (trimethylene carbonate) (PTMC-MA) and prepared a bioresin composed of PTMC-MA and hydroxyapatite nanoparticles (Nano-HAP). Furthermore, SLA was employed to fabricate biodegradable composite bone scaffolds that exhibited a hierarchical structure resembling the native bone. The scaffolds performed interconnected pores with high porosity, and Nano-HAP was uniformly distributed within the scaffold (Figure 9e). Human bone marrow mesenchymal stem cells (hBMSCs) were seeded on the surfaces of PTMC-MA scaffolds with and without nano-HAP, respectively. After in vitro cultivation, the DNA content of hBMSCs on the nano-HAP-containing scaffold surface was significantly higher than that on the scaffold without nano-HAP. This indicated that nano-HAP promoted cell adhesion and proliferation on the scaffold surface. The scaffolds were implanted into rabbit skull defects, and samples were collected at 0, 3, and 6 weeks post-surgery for micro-CT and histological examination of new bone growth at the defect site (Figure 9f). The results revealed that the rabbits implanted with PTMA-MA scaffolds containing 40% nano-HAP exhibited the highest degree of vascularization at the lesion site, effectively inducing the formation of new bone tissue in rabbits.

Cartilage is a type of connective tissue that is typically found in various parts of the human body, such as joints, ears, nose, and trachea. It is a tough yet flexible tissue characterized by its elasticity and resistance to compression, providing support and cushioning in joints [237]. Its primary functions in the body include reducing friction between joints, maintaining the shape of organs, and supporting the formation of the skeletal structure during growth and development [238]. However, unlike bone tissue, cartilage lacks a vascular blood supply, which means its regenerative capacity is relatively limited once damaged. Cartilage injuries or degeneration can lead to pain and impaired mobility, making research in cartilage repair and regeneration very important [239]. GelMA and HAMA have been widely employed in the fabrication of scaffolds encapsulated with various cell types relevant to cartilage regeneration, including articular chondrocytes and chondroprogenitor cells [240,241]. Sun et al. [242] bioprinted a biodegradable [poly-D, L-lactic acid/polyethylene glycol/poly-D, L-lactic acid (PDLLA − PEG)]/HA hydrogel encapsulated with hADSCs by using DLP. The encapsulated cells within the structure effectively underwent the process of chondrogenic differentiation. Bernal et al. [243] used GelMA containing articular cartilage progenitor cells (ACPCs) as bioink and successfully printed a human-sized meniscus in just a matter of seconds using CAL (Figure 9g). The printing precision of these structures far surpassed that of extrusion printing and DLP printing. After 28 days of in vitro culture, the printed meniscus structure showed a significant enhancement in the metabolism of ACPCs. The distribution of glycosaminoglycans (GAGs) and Type I collagen in the scaffolds was uniform and highly abundant, while Type II collagen content was relatively lower (Figure 9h). These results indicated that the structures printed using CAL provide favorable conditions for cell growth and proliferation, holding promising applications in the fields of tissue engineering and regenerative medicine.

Bones and cartilage serve as vital load-bearing tissues within the human body. For 3D bioprinting of bone and cartilage tissue structures, though the bioprinted constructs exhibited improved macroscopic and microscopic features, achieving mechanical compatibility with the damaged sites presents a significant challenge. The integration of native bone or cartilage with the constructs is also a big challenge. Therefore, a crucial development direction is the bioprinting and construction of integrated bone-cartilage tissues and grafts.

## 6. Conclusions

VP has been widely utilized in the fields of tissue engineering and regenerative medicine due to its exceptional spatiotemporal control over bioresin, which enables the fabrication of complex 3D hierarchical structures closely resembling human tissues and organs. Its advantages include rapid printing speed, high printing integrity, and excellent resolution. Various techniques, such as SLA, DLP, CLIP, HARP, and CLA, have been employed in the fabrication of scaffolds for the regeneration of different tissues, including the liver, skin, bone, cartilage, and more. Over the past few decades, significant efforts have been made to develop photoactive biomaterials with high biocompatibility and appropriate biodegradability. Natural biomaterials such as gelatin, hyaluronic acid, chitosan, sodium alginate, and their derivatives are among the most widely used due to their excellent biocompatibility, non-immunogenicity, and biodegradability. Synthetic biomaterials, while less biocompatible, offer better mechanical strength. Although research on VP-based bioprinting of human tissues, including the liver, skin, bone, etc., has been widely reported, the complete construction of transplantable tissues has not yet been achieved. To address this problem, several challenges need to be overcome.

1. VP is capable of printing structures with unprecedented resolution, especially in TPP. However, the higher the resolution and the larger the size, the slower the printing speed. Therefore, developing VP techniques with high resolution, high printing speed, and high throughput is the future direction.
2. The structure of different human tissues is highly complex, consisting of a variety of materials and various types of cells. One prerequisite for achieving VP bioprinted tissue that perfectly replicates human tissue is enabling the use of multiple materials in the fabrication process. However, the bioresin used in VP is stored in a vat, making it more challenging to realize multimaterial printing in VP compared to extrusion bioprinting and inkjet bioprinting. Developing multimaterial VP-based bioprinting is also a future trend.
3. Materials suitable for VP-based bioprinting have been extensively developed in the past decades. However, the variety of biomaterials suitable for VP-based bioprinting is still insufficient. The bioresin used in VP-based bioprinting needs to have low viscosity, which can lead to the sedimentation of encapsulated cells. Moreover, bioresins used in VP need to be transparent so that light can penetrate the bioresin and initiate photopolymerization. However, highly transparent biomaterials for bioresins are relatively limited. Thus, another future trend is the development of materials for bioresins and universal bioresin toolboxes that address the challenge of cell encapsulation while managing viscosity.

## Data Availability

Not applicable.
